# Therapy process factors in early psychosis: The effect of working alliance on clinical symptoms and cognitive biases in Ultra High Risk young people

**DOI:** 10.1016/j.schres.2025.07.031

**Published:** 2025-08-06

**Authors:** Paulina Bagrowska, Cassandra Wannan, Andrea Polari, Hok Pan Yuen, Paul Amminger, Melissa Kerr, Jessica Spark, Nicky Wallis, Martha Shumway, Cameron Carter, Lisa Dixon, Tara A. Niendam, Rachel Loewy, Patrick McGorry, Barnaby Nelson

**Affiliations:** a Experimental Psychopathology Lab, Institute of Psychology, Polish Academy of Sciences, Warsaw, Poland; b Orygen, The National Centre of Excellence in Youth Mental Health, The University of Melbourne, Parkville, Victoria, Australia; c Centre for Youth Mental Health, The University of Melbourne, Parkville, Victoria, Australia; d Orygen Specialist Program, Melbourne, Victoria, Australia; e Department of Psychiatry and Behavioral Sciences, University of California, San Francisco, United States of America; f Department of Psychiatry and Behavioral Sciences, University of California, Davis, Sacramento, United States of America; g Department of Psychiatry, Columbia University, New York, New York, United States of America

**Keywords:** UHR, Ultra-High-Risk, Psychosis, Psychotic disorders, Working alliance, Therapeutic alliance, Cognitive biases

## Abstract

Approximately 25 % of individuals at Ultra High Risk (UHR) transition to a full psychotic disorder. Cognitive biases are thought to play a role in this risk, and improvements in cognitive biases have been associated with better clinical outcomes. Early intervention is crucial, yet no therapeutic approach has proven superior, and treatment adherence remains a challenge. Therapeutic alliance, shown to enhance adherence and clinical outcomes, might also influence depression and cognitive biases, but its role in these domains among UHR patients remains unclear. A total of 202 UHR participants (59.4 % females, mean age 17.4), taking part in the sequential multiple-assignment randomized trial (SMART), completed questionnaires assessing cognitive biases, attenuated psychotic symptoms, quality of life, general and social functioning, depression, and overall psychopathology at baseline, and at 6- and 12-month follow-ups. Additionally, both patients and their corresponding therapists rated working alliance following the 6- and 12-month treatment periods. The results revealed that patient-rated therapeutic alliance significantly predicted improvements in attenuated psychotic symptoms, cognitive biases, general psychopathology, social functioning, and quality of life, with the strongest effects observed when measured 6 months into treatment. The relationship between working alliance and decreases in attenuated psychotic symptoms, as well as between working alliance and reduction in general psychopathology, turned out to be mediated by improvements in cognitive biases. Therapist-rated working alliance did not significantly predict clinical improvements, showing only a minor association with quality of life. Strengthening the therapeutic alliance and prioritizing cognitive bias modification early in interventions for UHR patients may improve treatment outcomes. Further research is needed to validate these findings.

## 1. Introduction

People at ultra-high risk (UHR) of developing psychosis experience early subthreshold symptoms, which, although they do not meet the criteria for full-blown psychotic disorders, are associated with a high level of distress and a number of negative mental health consequences. This putative prodromal period, which can last from weeks to years (Salazar de Pablo et al., 2021), is characterized by functional and social impairment, the occurrence of subclinical, attenuated psychotic symptoms, and the presence of additional comorbid psychiatric disorders. This stage often precedes the development of more severe psychotic states, with approximately 25 % of individuals progressing to develop a threshold psychotic disorder within 3 years, with enhanced risk persisting for up to 10 years (Salazar de Pablo et al., 2021). Importantly, UHR individuals who do not transition to psychosis remain at significant risk for continued attenuated psychotic symptoms, as well as other persistent or recurrent conditions such as mood and anxiety disorders (Lin et al., 2015; Solmi et al., 2023). The awareness that early signs may precede the onset of the disease has drawn attention to the need to identify people who may be in such a risk group. Early recognition of risk states is crucial as it allows for timely intervention to alleviate ongoing difficulties and reduce the likelihood of progression to not only full-blown psychotic disorder but also other serious mental health conditions. Indeed, over the last two decades, a number of randomized controlled trials (RCTs), systematic reviews, and meta-analyses on the efficacy of various therapeutic interventions for UHR patients have been published (Mei et al., 2021). The results do not fully determine the superiority of one type of treatment over another. Nonetheless, they indicate significant benefits and efficacy in reducing the severity of clinical symptoms and delaying (or preventing) the transition to psychosis (Devoe et al., 2020; McGorry et al., 2021, 2023; Stafford et al., 2013), especially following psychosocial interventions such as cognitive behavioral therapy (CBT) (Hutton and Taylor, 2014; Mei et al., 2021; Morrison et al., 2004).

The current research presents secondary analyses from the Staged Treatment in Early Psychosis (STEP) study (Nelson et al., 2018; McGorry et al., 2023), a sequential multiple assignment randomized trial (SMART) conducted within the clinical program at Orygen, Melbourne, Australia. The trial recruited young individuals aged 12 to 25 years meeting the UHR criteria. The intervention was delivered in three steps: Step 1 involved Support and Problem Solving (SPS); Step 2 compared SPS with Cognitive Behavioural Case Management (CBCM); and Step 3 compared CBCM combined with antidepressant medication versus CBCM with placebo. Participants who did not respond to treatment at a given step progressed to the next stage (please refer to the Methods section for further details on the study design). The results of the STEP study showed no significant differences in symptom and functional outcomes, as well as transition to psychosis rates between the treatment modalities used. However, the results demonstrated overall functional and symptomatic improvement following the intervention. For instance, it has been shown that decrease in cognitive biases six months into treatment has been associated with greater improvements in general psychopathology, depressive symptoms, and quality of life among UHR patients (McGorry et al., 2023). Although this effect did not significantly differ between treatment groups (which included CBT-based interventions and support and problem-solving), it is essential to highlight that cognitive biases can be a crucial mediator or even a direct target of treatment for subthreshold psychotic symptoms. This is consistent with a recent meta-analysis suggesting that changes in cognitive biases in response to treatment (which may be one of the key mechanisms at work in CBT-based interventions) should be considered as the primary outcome of treatment effectiveness studies to better understand how modification of cognitive biases leads to improvements in psychotic symptoms (Sauvé et al., 2020 ). The cognitive model of psychosis suggests that, in light of pre-existing vulnerabilities (i.e., biopsychosocial predispositions), disturbed cognitive processes lead to the development of anomalous experiences, which, under the influence of negative emotional states, contribute to the formation of psychotic symptoms and thus to the onset or maintenance of psychotic disorders (Garety et al., 2001; Howes and Murray, 2014). Distorted cognitive processes include cognitive biases, such as jumping to conclusions (JTC) and belief inflexibility (reasoning biases), attention to threat and aberrant salience (attentional biases), as well as external attributions and personalizing (attributional biases) (Garety and Freeman, 1999; Livet et al., 2020). Recent systematic reviews and meta-analyses revealed significant associations between subclinical psychotic symptoms and cognitive biases in individuals at high risk of developing psychosis, and, in line with the cognitive model of psychosis, emphasized the contribution of cognitive biases to the emergence and maintenance of both positive and negative symptoms (Gawęda et al., 2024; Livet et al., 2020).

It is noteworthy that the STEP study reported a substantial rate of treatment discontinuation: 17.8 % at Step 1 (61 of 342), 47.7 % at Step 2 (134 of 281), and 38.4 % at Step 3 (53 of 138) (McGorry et al., 2023), which, although represents a common challenge in intervention and psychotherapy research, highlights the need for further investigation into underlying factors within the therapeutic processes. In the absence of clear evidence of the superiority of one type of treatment over another, the question arises as to what other factors associated with the therapeutic intervention may have a positive impact and contribute to patient engagement/drop out. One of the primary aspects influencing treatment outcomes, which has long been described in the literature and is still the subject of research, is the quality of the relationship between therapist and client, i.e., working alliance (Baier et al., 2020; Horvath and Symonds, 1991). The definition of a working alliance consists of three main processes: mutual agreement on the *goals* of therapy and assigned *tasks* to achieve these goals, and a high-quality client-therapist relationship (*bond*) based on trust and acceptance (Bordin, 1979). It has been suggested that the quality of the therapist-patient relationship, which may be particularly challenging in the case of psychotic patients, plays a pivotal role in treatment outcomes (McCabe and Priebe, 2008). A recent meta-analysis examining the role of the working alliance in treatment response in individuals with schizophrenia and early psychosis found that a better therapeutic alliance was associated with greater treatment engagement and contributed to improvements in positive and negative symptoms (Browne et al., 2021). In addition, a stronger therapeutic alliance has previously been found in a first episode psychosis cohort to be associated with improved mental health recovery, psychological well-being, quality of life, total symptoms, as well as negative and disorganized symptoms after treatment (Browne et al., 2019). Another study also found significant associations between working alliance and negative and disorganized symptoms, as well as social functioning in patients with first-episode psychosis (FEP) (Melau et al., 2015). The findings indicated that a stronger working alliance was related to fewer negative and disorganized symptoms and better social functioning. In addition, the authors suggested that the working alliance may not be the sole keystone in positive treatment outcomes, but may be an essential element in influencing therapy adherence, which builds a foundation for the development of self-efficacy, which could potentially be a significant mediator in the relationship between the working alliance and clinical outcomes.

However, research findings indicating the positive impact of a good therapeutic relationship on symptom improvement in people suffering from psychosis are not entirely conclusive. In particular, the role of therapeutic alliance in other important therapy targets beyond psychotic symptoms, such as general functioning or depression, is less clear. For instance, a recent review and meta-analysis (Bourke et al., 2021) comprising participants diagnosed with either affective or non-affective psychosis found that the therapeutic relationship was significantly associated with a reduction in global and psychotic symptoms and had a positive impact on engagement in therapy. In contrast, no or very limited evidence of an association with depressive symptoms or social functioning was observed. Existing research also lacks evidence on the potential impact of working alliance on the cognitive biases that may contribute to the onset and maintenance of psychotic symptoms. Moreover, the role of working alliance in the UHR group, which is characterized by severe, although still milder, symptoms than patients with first-episode psychosis or chronic schizophrenia, is yet to be examined. This is particularly important as early intervention is crucial for patients in UHR for delaying or preventing the development of psychosis, and further research into factors that may improve treatment adherence and outcomes is required.

Against this background, the current study aimed to investigate the predictive role of working alliance on clinical outcomes (clinical symptoms, global and social functioning, quality of life) and cognitive biases in a UHR sample. Additionally, the study aimed to test the mediating role of cognitive biases improvement in the relationship between working alliance and attenuated psychotic symptoms, as well as between working alliance and general psychopathology symptoms (regardless of treatment type delivered in the context of an intervention trial).

## 2. Methods

### 2.1. Study design

A detailed study design and methods description was recently reported (McGorry et al., 2023; Nelson et al., 2018). Briefly, the primary aim of this study was to examine the effectiveness of a sequential multiple assignment randomized trial (SMART) in UHR individuals. The sequential intervention strategy consisted of three stages: Support and Problem Solving (SPS), Cognitive Behavioral Case Management (CBCM), and antidepressant medication. Participants who did not respond to the intervention during a step progressed to the next stage of treatment. Response to the intervention was evaluated based on remission status, as reflected by sustained functional (primary outcome – Global Functioning: Social and Role scales (Cornblatt et al., 2007)) and symptomatic (based on the Comprehensive Assessment of At-Risk Mental States (Yung et al., 2005)) improvement across treatment steps.

In step 1 (6 weeks), open-label SPS was provided to all participants. SPS covers primary supportive counselling and problem-solving strategies within a positive psychology framework. The SPS sessions lasted between 30 and 50 min and took place weekly to fortnightly. Participants who responded to Step 1 were randomly assigned to monthly SPS sessions or mental health and risk monitoring at 3, 6, 9, and 12 months after treatment to ensure relapse prevention. Participants who did not respond to SPS in Step 1 were randomly assigned to one of two treatment groups in Step 2, which included CBCM and SPS for 20 weeks. Cognitive Behavioral Case Management is a psychosocial intervention consisting of cognitive behavioral therapy (CBT) techniques delivered within a case management framework that addresses ongoing practical and social difficulties. The CBCM sessions were 30 to 50 min, delivered weekly or as needed. Those who remitted were further randomized to SPS or monitoring, and those who did not respond to treatment at this stage were randomly assigned to one of two subsequent intervention groups in Step 3, which included continuing CBCM in combination with an antidepressant medication (fluoxetine) or CBCM plus placebo for 26 weeks. During Step 3, at 12 weeks, participants who showed no response or worsened could, through a shared decision-making process with their treating psychiatrist, choose to either continue their treatment as randomized, adjust the dosage, or add low-dose antipsychotics (quetiapine or aripiprazole) or long-chain ω−3 fatty acids. Randomization to treatment allocation was computer-generated and managed by independent personnel. Assessors evaluating remission criteria were blinded to treatment allocation, and in Step 3, both assessors and participants were blinded to treatment allocation.

The Melbourne Health Human Research Ethics Committee approved the study (no. 2015.173).

### 2.2. Participants

This study is a secondary analysis from a 12-month, sequential multiple assignment randomized trial (SMART) conducted between 2016 and 2019 at Orygen, Melbourne, Australia (a part of The Staged Treatment in Early Psychosis [STEP] project) (Nelson et al., 2018; McGorry et al., 2023).

A total of 342 participants took part in the main study (McGorry et al., 2023). However, data on the working alliance was only available for 202 individuals, making this the final sample size in the current work. Participants included in the study were aged 12 to 25 years, English-speaking, able to give informed consent, and meeting the criteria of at least one of the UHR for psychosis groups (Trait Vulnerability, APS, BLIPS, see Table 1 in Supplementary materials or Nelson et al. (2018) for details). Exclusion criteria covered a history of a psychotic episode lasting more than one week, attenuated psychotic symptoms occurring only during acute intoxication, neurological and intellectual disorders, antipsychotic medication exposure above a certain threshold, current use of antidepressant medications, and physical illness. Participants were recruited from Orygen’s primary care (headspace clinics) and specialist clinics (Orygen Specialist Program), which provide mental health care for 12–25-year-olds. Additionally, 39 therapists were assigned to study participants, providing a total of 151 therapist working alliance assessments.

### 2.3. Measures

*Working Alliance Inventory* (WAI) (Horvath and Greenberg, 1989) was used to assess the quality of the therapeutic alliance 6 and 12 months into treatment. Specifically, the participants completed the client version (WAI-C), and their corresponding therapists completed the therapist version (WAI-T). We used a shortened version of the WAI (Hatcher and Gillaspy, 2006), with items rated on a 5-point Likert scale (1 to 5). The WAI-C consisted of 12 items (score range: 12 to 60), and the WAI-T consisted of 10 items (score range: 10 to 50). Higher scores on both versions indicate a stronger working alliance.

A comprehensive battery of validated instruments was used. This included the *Comprehensive Assessment of At-Risk Mental States* (CAARMS) (Yung et al., 2005) to evaluate attenuated psychotic symptoms (higher scores indicate higher levels of attenuated psychotic symptoms), *Brief Psychiatric Rating Scale* (BPRS) (Overall and Gorham, 1962) to assess the severity of general psychopathology symptoms, such as depression, anxiety and hallucinations (higher scores indicate higher levels of general psychopathology); *Davos Assessment of Cognitive Biases Scale* (DACOBS) (van der Gaag et al., 2013) to assess various cognitive biases (higher scores indicate higher levels of cognitive biases); *Montgomery-Åsberg Depression Rating Scale* (MADRS) (Montgomery and Asberg, 1979) to assess depressive symptoms (higher scores indicate higher levels of depressive symptoms); *Global Functioning: Social and Role scales* (GFS; GFR) (Cornblatt et al., 2007) for global functioning assessment (higher scores indicate better global functioning); *Scale for the Assessment of Negative Symptoms* (SANS) (Andreasen, 1989) to assess the severity of negative symptoms (higher scores indicate higher levels of negative symptoms); *Social and Occupational Functioning Assessment Scale* (SOFAS) (Goldman et al., 1992) to measure social functioning (higher scores indicate better functioning); and *Assessment of Quality of Life* (AQoL) (Hawthorne et al., 1999) to assess the health-related life quality (higher scores indicate lower quality of life). For full details, see Nelson et al. (2018).

### 2.4. Statistical analysis

Statistical analyses were carried out using SPSS 29. First, a two-tailed Pearson’s correlation analyses were performed to investigate the association between outcome variables measured at baseline and working alliance measured at 6 and 12 months into treatment. Linear regression analyses were performed to examine the association between working alliance and all individual outcome measures (i.e., change scores), adjusted for age, gender, type of treatment and baseline measure of a given variable. The change score was calculated by subtracting the baseline value from the 6-month (T1 - T0) or 12-month (T2 - T0) score. Furthermore, simple mediation analyses examining the role of 6-month change in cognitive biases in the relationship between working alliance and the 6-month change in attenuated positive psychotic symptoms, as well as in the relationship between working alliance and the 6-month change in general psychopathology were conducted using model 4 in the PROCESS macro (Preacher and Hayes, 2004), following the bootstrapping procedure with 5000 resamples. Likewise, this model controlled for age, gender, treatment type, and baseline measures of cognitive biases and severity of attenuated psychotic symptoms or general psychopathology.

## 3. Results

The study comprised a total of 342 treatment-seeking individuals, all of whom met UHR criteria. The primary focus of the current analysis was working alliance, so analyses were conducted on the subset of participants who completed the Working Alliance Inventory (WAI). Cases in which only the therapist or only the patient completed the WAI were included in analysis.

Finally, the results are based on data collected from 202 young people, of whom 120 were female (59.4 %) and 82 were male (40.6 %), aged between 12 and 24 years (*M* = 17.4; *SD* = 3.1). With the exception of baseline SOFAS (*p* = 0.03), on which participants who completed the WAI had slightly higher scores (*M* = 57.8, *SD* = 11.7) than those who did not (*M* = 55.1, *SD* = 11.3), no significant differences were observed on the remaining outcome variables at baseline between WAI completers and non-completers. A total of 202 participants completed the WAI-C, which had a mean total score of 45.1 (*SD* = 8.7; score range 19–60). Additionally, 151 WAI-T assessments were completed by therapists, with a mean total score of 39.5 (*SD* = 5.6; score range 26–50) at 6-month follow-up. In terms of 12-month follow-up, 82 participants completed the WAI-C (*M* = 45.2; *SD* = 9.5; score range 19–60), and 56 WAI-T assessments were completed by therapists (*M* = 41.5; *SD* = 5.2; score range 26–50). No statistically significant differences were observed in either the WAI-C or WAI-T scores between the 6- and 12-month follow-up periods. Sample characteristics can be found in Table 1.

### 3.1. Correlation analyses

The results of the correlation analysis between baseline measurements and working alliance assessed both 6 and 12 months into treatment are shown in Table 2. The results revealed significant correlations between WAI-C rated at 6-month follow-up and baseline measures of BPRS, MADRS, SANS, and AQoL. A stronger working alliance, as assessed by participants, was associated with lower baseline levels of general psychopathology, depressive symptoms, and negative symptoms, as well as more positive quality of life. WAI-C rated at 12-months only correlated significantly with baseline BPRS and GFR, suggesting that a stronger working alliance was associated with lower baseline general psychopathology and better general functioning. In terms of working alliance rated by therapists, WAI-T rated at 6-months correlated with baseline BPRS and SANS, indicating that a stronger alliance was associated with lower baseline general psychopathology and negative symptoms. At 12-months, WAI-T was correlated with baseline BPRS, GFR, and SANS, suggesting that a stronger alliance was related to lower psychopathology, better general functioning, and less severe negative symptoms.

### 3.2. Linear regression analyses

Table 3 presents the results of linear regression analyses performed to examine the associations between the participants’ assessment of the working alliance (WAI-C) and changes in outcomes following the intervention. Table 4 presents the results of linear regression analyses examining the associations between the working alliance rated by therapists (WAI-T) and changes in outcome variables.

The results revealed that the WAI-C assessed 6 months into treatment was significantly associated with 6-month changes in cognitive biases (DACOBS), attenuated positive psychotic symptoms (CAARMS), general psychopathology (BPRS), social functioning (SOFAS) and quality of life (AQol). In addition, the WAI-C assessed at the 6-month follow-up proved to be significantly associated with 12-month changes (from baseline to 12 months) in BPRS, MADRS, SANS, AQoL, and marginally DACOBS (*p* = 0.07).

The WAI-T was found to be significantly associated only with the 6-month changes in the quality of life (AQoL) when assessed at the 6-month follow-up.

### 3.3. Mediation analysis

**Fig. 1.**
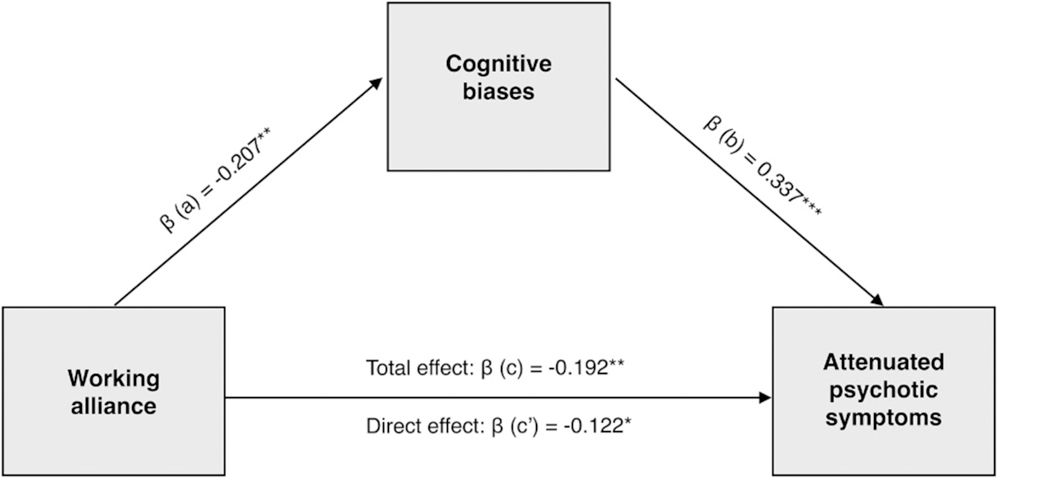
The mediating effect of changes in cognitive biases on the relationship between working alliance and changes in attenuated psychotic symptoms.

Fig. 1 presents the results of a mediation analysis aimed at exploring the mediating effect of changes in cognitive biases 6-months into treatment on the relationship between participant-rated working alliance (WAI-C at 6 months) and 6-months into treatment change in attenuated psychotic symptoms (CAARMS).

The standardized total effect of working alliance on 6-month change in attenuated psychotic symptoms was statistically significant (*β* = − 0.19, 95 % CI = _−_ 0.76 to − 0.16, *p* < 0.01). The bootstrapping estimate revealed a significant standardized indirect effect of working alliance on 6-month change in attenuated psychotic symptoms through 6-month change in cognitive biases (*β* = − 0.07, 95 % CI = − 0.122 to − 0.025). The standardized direct effect of working alliance on change in attenuated psychotic symptoms remained significant (*β* = − 0.122, 95 % CI = − 0.577 to − 0.011, *p* = 0.042), which means that the mediation is complementary (Zhao et al., 2010). The total effect explained 31.53 % of the variance in attenuated psychotic symptoms, while the mediated model explained 41.48 % of the variance. Covariates such as gender (*p* = 0.02), age (*p* > 0.05), treatment type (*p* > 0.05), and baseline measures of cognitive biases (*p* = 0.002) and attenuated psychotic symptoms (*p* < 0.001) were included in the model.

The mediation analysis exploring the mediating effect of a 6-month change in cognitive biases between therapist-rated working alliance (WAI-T) and a 6-month change in attenuated psychotic symptoms revealed no significant total, direct, or indirect effects.

**Fig. 2.**
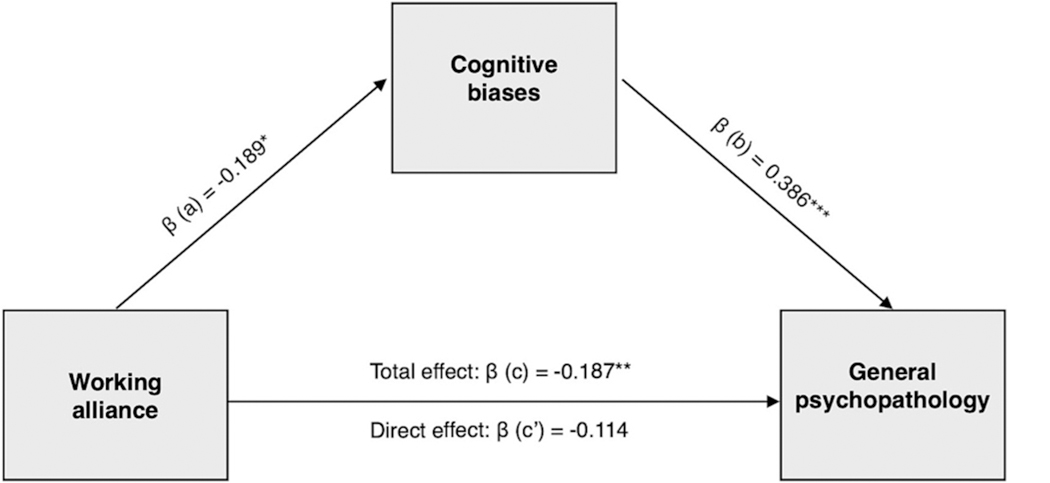
The mediating effect of changes in cognitive biases on the relationship between working alliance and changes in general psychopathology symptoms.

Fig. 2 presents the results of a mediation analysis aimed at exploring the mediating effect of changes in cognitive biases 6 months into treatment on the relationship between participant-rated working alliance (WAI-C at 6 months) and a change in general psychopathology (BPRS) between baseline and 6 months.

The standardized total effect of working alliance on 6-month change in general psychopathology was statistically significant (*β* = − 0.187, 95 % CI = _−_ 0.319 to − 0.049, *p* < 0.01). The bootstrapping estimate revealed a significant standardized indirect effect of working alliance on 6-month change in general psychopathology through 6-month change in cognitive biases (*β* = − 0.07, 95 % CI = − 0.131 to − 0.02). The standardized direct effect of working alliance on change in general psychopathology was not statistically significant (*β* = − 0.114, 95 % CI = − 0.238 to 0.014, *p* = 0.081), which means that the mediation is indirect- only. The total effect explained 21.45 % of the variance in general psychopathology, while the mediated model explained 34.47 % of the variance. Covariates such as gender (*p* >0.05), age (*p >* 0.05), treatment type (*p* > 0.05), and baseline measures of cognitive biases (*p* > 0.05) and general psychopathology (*p* < 0.001) were included in the model.

## 4. Discussion

This study aimed to investigate the relationship between working alliance in psychotherapy and clinical and functional outcomes in young people at clinically high risk for developing psychosis.

Our findings highlight the important role of a strong therapeutic alliance in driving positive treatment outcomes. Specifically, a strong participant-rated working alliance at six months into treatment was significantly associated with improvements in attenuated psychotic symptoms, general psychopathology, cognitive biases, social functioning, and perceived quality of life. These effects were not limited to the short term – six-month alliance ratings were also associated with 12-month improvements in depressive symptoms, general psychopathology, negative symptoms, and quality of life. Although the association between working alliance and cognitive biases at 12 months did not reach statistical significance, the effect sizes were similar to those observed at six months. This suggests that the absence of significance is likely due to reduced sample size at the 12-month follow-up, rather than a lack of true association. These findings should therefore be interpreted with caution. Taken together, the results support the idea that a strong therapeutic relationship may be a critical factor driving improvements across a broad range of clinical outcomes in individuals at ultra-high risk for psychosis.

Interestingly, the therapist-rated working alliance did not appear to be significantly associated with almost any (other than a weak correla-tion with quality of life) changes in treatment outcomes. Although WAI- C and WAI-T were significantly and positively correlated at both 6- and 12-month follow-ups (see results in supplementary materials), they did not have a similar effect on treatment response. Therapist ratings of the working alliance were, however, associated with lower general psy-chopathology symptoms, lower negative symptoms, and better global functioning of participants at baseline, which is in line with results ob-tained by Shattock et al. (2018)in their systematic review. It is plausible that therapists tend to report stronger therapeutic alliance with clients who exhibit lower levels of symptom severity. A similar pattern was observed for participant ratings, which may suggest that a high-quality therapeutic alliance has greater potential for favorable outcomes when accompanied by higher levels of participants’ current abilities and cognitive insight (i.e., lower psychopathology, better global func-tioning). This may suggest that clinicians should prioritize the estab-lishment of a strong therapeutic alliance, taking into account patients’ current capabilities and levels of cognitive insight. Communication with patients exhibiting varying symptom severity (Priebe et al., 2010) should be tailored to meet their individual needs and set goals, which is one of the key components of working alliance (Browne et al., 2021). It is therefore recommended that further research explore strategies for building a strong therapeutic alliance, with a particular focus on patients who experience communication difficulties, pronounced negative symptoms, or poor overall functioning. Additionally, alliance-building may serve not only as a supportive process but as a mechanism that actively drives improvements in clinical outcomes, and future in-terventions may benefit from explicitly targeting alliance as a thera-peutic goal. However, given the reduced sample size for the WAI-T, which may have limited the ability to detect significant effects, these findings should be interpreted with caution and warrant replication in future studies with larger samples.

The first working alliance assessment, conducted six months into treatment, was associated with changes in cognitive biases and, weaker yet still significant, changes in attenuated psychotic symptoms. The re-sults of the mediation analysis supported our hypothesis that improve-ments in cognitive biases partially mediated the link between therapeutic alliance and reductions in subclinical symptoms. In other words, a stronger therapeutic relationship, as perceived by the partici-pant, may enhance their ability to modify cognitive biases, which in turn contributes to improvements in other clinical outcomes. The analysis revealed both an indirect effect of cognitive bias change and a direct effect of therapeutic alliance on the decrease in attenuated psychotic symptoms, indicating a complementary mediation effect. This suggests that while therapeutic alliance directly impacts clinical change, the improvement in cognitive biases further enhances this effect. It also suggests that there may be other factors (mediators) that contribute to explaining this relationship, which would be worth exploring in future research. In addition, a second mediation analysis examining the rela-tionship between therapeutic alliance and improvement in general psychopathology (as measured by the BPRS) revealed a full mediation effect. In this case, the association between working alliance and general symptom reduction was entirely accounted for by improvements in cognitive biases. This suggests that changes in cognitive biases may be a key mechanism through which the therapeutic relationship influences broader psychopathological outcomes, highlighting the central role of cognitive processes.

Interestingly, the therapist-rated working alliance did not appear to be significantly associated with almost any (other than a weak correlation with quality of life) changes in treatment outcomes. Although WAI-C and WAI-T were significantly and positively correlated at both 6- and 12-month follow-ups (see results in supplementary materials), they did not have a similar effect on treatment response. Therapist ratings of the working alliance were, however, associated with lower general psychopathology symptoms, lower negative symptoms, and better global functioning of participants at baseline, which is in line with results obtained by Shattock et al. (2018) in their systematic review. It is plausible that therapists tend to report stronger therapeutic alliance with clients who exhibit lower levels of symptom severity. A similar pattern was observed for participant ratings, which may suggest that a high-quality therapeutic alliance has greater potential for favorable outcomes when accompanied by higher levels of participants’ current abilities and cognitive insight (i.e., lower psychopathology, better global functioning). This may suggest that clinicians should prioritize the establishment of a strong therapeutic alliance, taking into account patients’ current capabilities and levels of cognitive insight. Communication with patients exhibiting varying symptom severity (Priebe et al., 2010) should be tailored to meet their individual needs and set goals, which is one of the key components of working alliance (Browne et al., 2021). It is therefore recommended that further research explore strategies for building a strong therapeutic alliance, with a particular focus on patients who experience communication difficulties, pronounced negative symptoms, or poor overall functioning. Additionally, alliance-building may serve not only as a supportive process but as a mechanism that actively drives improvements in clinical outcomes, and future interventions may benefit from explicitly targeting alliance as a therapeutic goal. However, given the reduced sample size for the WAI-T, which may have limited the ability to detect significant effects, these findings should be interpreted with caution and warrant replication in future studies with larger samples.

The first working alliance assessment, conducted six months into treatment, was associated with changes in cognitive biases and, weaker yet still significant, changes in attenuated psychotic symptoms. The results of the mediation analysis supported our hypothesis that improvements in cognitive biases partially mediated the link between therapeutic alliance and reductions in subclinical symptoms. In other words, a stronger therapeutic relationship, as perceived by the participant, may enhance their ability to modify cognitive biases, which in turn contributes to improvements in other clinical outcomes. The analysis revealed both an indirect effect of cognitive bias change and a direct effect of therapeutic alliance on the decrease in attenuated psychotic symptoms, indicating a complementary mediation effect. This suggests that while therapeutic alliance directly impacts clinical change, the improvement in cognitive biases further enhances this effect. It also suggests that there may be other factors (mediators) that contribute to explaining this relationship, which would be worth exploring in future research. In addition, a second mediation analysis examining the relationship between therapeutic alliance and improvement in general psychopathology (as measured by the BPRS) revealed a full mediation effect. In this case, the association between working alliance and general symptom reduction was entirely accounted for by improvements in cognitive biases. This suggests that changes in cognitive biases may be a key mechanism through which the therapeutic relationship influences broader psychopathological outcomes, highlighting the central role of cognitive processes.

It remains a question – and a worthwhile area for further investigation - whether the response to cognitive bias treatment manifests more rapidly compared to other mental health difficulties. Indeed, it has been proposed that modifying cognitive processes first could exert a more substantial impact on subsequent functional or clinical outcomes (Jones and Sharpe, 2017), such as the beneficial influence of improving cognitive biases on positive psychotic symptoms (Sauvé et al., 2020 ). Given their crucial role in clinical and subclinical psychotic symptoms, as well as their significant association with the working alliance, targeting cognitive biases directly while continuously monitoring and strengthening the therapeutic relationship could be possibly considered a first-line intervention for UHR individuals. However, while the early targeting of cognitive biases remains a plausible mechanism of change, an alternative explanation, more consistent with the current study’s findings, warrants consideration. In the present study, participants received two different psychotherapeutic interventions, only one of which (i.e., CBCM) directly addressed cognitive biases. As no significant differences in outcomes were observed between intervention types, it is also plausible that a strong therapeutic alliance itself, independent of specific techniques targeting cognitive biases, may have resulted in changes to cognitive processing. A positive therapeutic relationship may increase a client’s openness to the therapist’s perspective and alternative interpretations of experiences, thereby fostering greater cognitive flexibility and indirectly weakening the influence of cognitive biases. This perspective aligns with the idea of the therapeutic alliance not merely as a supportive aspect in treatment, but as an active mechanism of change in its own right.

While a significant proportion of the reported results demonstrated statistical significance, there are several limitations of this study which should be considered while interpreting our results. In this study, fewer WAI assessments were completed by therapists than by participants, so the results should be interpreted with caution with this aspect in mind. Moreover, a substantial proportion of participants did not complete the working alliance assessment, resulting in a notable amount of missing data and the necessity to conduct analyses on a sample that is considerably smaller than the total number of participants involved in the trial. Importantly, the only significant difference observed between participants who completed the WAI and those who did not was in social and occupational functioning (SOFAS), with completers demonstrating slightly higher baseline functioning. This difference may be indicative of a selection bias and should be considered a limitation of the current analysis. The absence of further investigation into the relationship between working alliance and treatment discontinuation represents a significant gap that should be addressed in future studies. An important limitation is the lack of measurement of the therapeutic alliance during the initial stages of treatment, hindering an accurate assessment of its impact on treatment outcomes. However, it is important to note that working alliance was not the primary focus of the main study (McGorry et al., 2023), hence, potentially important aspects allowing for a broader understanding might have been missed. Future research on working alliance in UHR treatment would benefit from the incorporation of qualitative methods, which could help delve into whether and which aspects of the therapeutic relationship are crucial for young people at risk for developing psychosis. This group may necessitate a unique approach compared to adults, emphasizing the need for a deeper understanding of how a valuable therapist-young person relationship can be established, as this may play a pivotal role in enhancing treatment engagement and adherence.

## 5. Conclusions

The role of therapeutic alliance in reducing the severity of cognitive biases, as a first step toward improving subsequent clinical symptoms in young people at ultra-high risk of developing psychosis has not previously been studied. The results of this analysis, although somewhat preliminary, suggest that both building a strong therapeutic relationship and reducing cognitive biases, which are sometimes referred to as an important early marker of psychotic disorders (Eisenacher and Zink, 2017), should be considered as one of the main and direct targets of therapeutic interventions for people in UHR. Importantly, the quality of the working alliance has been shown to account for a substantial amount of the variance in both attenuated psychotic symptoms, as well as general psychopathology improvement. However, further research is necessary to confirm and extend the results of our study.

## Supplementary Material

1

## Figures and Tables

**Table 1:** Baseline sample characteristics (n = 202).

	M ± SD

Age	17.4 ± 3.1
Female, no. (%)	120 (59.4 %)
Male, no. (%)	82 (40.6 %)
UHR – trait vulnerability, no. (%)	21 (10.4 %)
UHR - APS, no. (%)	199 (98.5 %)
UHR - BLIPS, no. (%)	5 (2.5 %)
Attenuated psychotic positive symptoms (CAARMS composite score)	35.3 ± 17.1
General psychopathology (BPRS total score)	44.2 ± 8.3
Cognitive biases (DACOBS total score)	168.0 ± 30.2
Depressive symptoms (MADRS total score)	22.6 ± 9.6
Global functioning: social (GFS)	6.6 ± 1.1
Global functioning: role (GFR)	6.5 ± 1.6
Negative symptoms (SANS total score)	17.8 ± 11.0
Social and occupational functioning (SOFAS)	57.8 ± 11.7
Quality of life (AQoL total score)	98.4 ± 17.8

*Note:* APS – Attenuated psychotic symptoms; BLIPS - Brief limited intermittent psychotic symptoms; CAARMS - Comprehensive Assessment of At-Risk Mental States (higher scores = more severe symptoms); BPRS - Brief Psychiatric Rating Scale (higher scores = more severe symptoms); DACOBS - Davos Assessment of Cognitive Biases Scale (higher scores = more severe biases); MADRS - Montgomery-Åsberg Depression Rating Scale (higher scores = more severe symptoms); GFS - Global Functioning: Social scale (higher scores = better functioning); GFR - Global Functioning: Role scale (higher scores = better functioning); SANS - Scale for the Assessment of Negative Symptoms (higher scores = more severe symptoms); SOFAS - Social and Occupational Functioning Assessment Scale (higher scores = better functioning); AQoL - Assessment of Quality of Life (higher scores = lower quality of life).

**Table 2:** Correlation matrix of baseline measures and working alliance assessed at 6 and 12 months into treatment.

	1	2	3	4	5	6	7	8	9	10	11	12

1. WAIC-6	.	.	.	.	.	.	.	.	.	.	.	.
2. WAIT-6	**0.272** ***	.	.	.	.	.	.	.	.	.	.	.
3. WAIC-12	**0.635** ***	0.202	.	.	.	.	.	.	.	.	.	.
4. WAIT-12	**0.337** *	**0.711** ***	**0.421** **	.	.	.	.	.	.	.	.	.
5.CAARMS	−0.075	−0.088	−0.015	−0.046	.	.	.	.	.	.	.	.
6.BPRS	−0.193**	−0.182*	−0.219*	−0.419**	**0.549** ***	.	.	.	.	.	.	.
7. DACOBS	−0.017	−0.162	−0.114	−0.131	**0.371** ***	**0.349** ***	.	.	.	.	.	.
8. MADRS	−0.225**	−0.020	−0.066	−0.205	**0.423** ***	**0.668** ***	**0.245** ***	.	.	.	.	.
9.GFS	0.072	0.140	0.088	0.066	−0.192**	−0.372***	−0.189**	−0.304***	.	.	.	.
10. GFR	0.083	0.101	**0.230** *	**0.287** *	−0.231***	−0.359***	−0.277***	−0.248***	0.391***	.	.	.
11. SANS	−0.174*	−0.162*	−0.181	−0.289*	**0.270** ***	**0.570** ***	**0.259** ***	**0.518** ***	−0.541***	−0.464***	.	.
12. SOFAS	0.039	0.071	0.129	0.205	−0.165*	−0.335***	−0.270***	−0.265***	**0.508** ***	**0.719** ***	−0.473***	.
13. AQoL	−0.215**	−0.110	−0.172	−0.233	**0.435** ***	**0.575** ***	**0.390** ***	**0.676** ***	−0.315***	−0.366***	**0.443** ***	−0.334***

*Note:* WAIC-6 - Working Alliance Inventory rated by participants at 6-month follow-up; WAIC-12 - Working Alliance Inventory rated by participants at 12-month follow-up; WAIT-6 - Working Alliance Inventory rated by therapists at 6-month follow-up; WAIT-12 - Working Alliance Inventory rated by therapists at 12-month follow-up; CAARMS - Comprehensive Assessment of At-Risk Mental States; BPRS - Brief Psychiatric Rating Scale; DACOBS - Davos Assessment of Cognitive Biases Scale; MADRS- Montgomery-Åsberg Depression Rating Scale; GFS - Global Functioning: Social scale; GFR - Global Functioning: Role scale; SANS - Scale for the Assessment of Negative Symptoms; SOFAS - Social and Occupational Functioning Assessment Scale; AQoL - Assessment of Quality of Life.

* <0.05.

** <0.01.

*** <0.001.

**Table 3:** Regression analysis summary. Association between **WAI-C total** [predictor] and outcome variables, controlled for gender, age, treatment type (Step 2 or Step 3) and baseline.

Outcome	*B*	95 % CI	*β*	*t*	*p*	adj *R*^*2*^

Association between WAI-C rated at 6-month and 6-month into treatment changes						
**CAARMS**	−**0.434**	−**0.72, −0.15**	−**0.181**	−**2.997**	**0.003**	**31 %**
**BPRS**	−**0.179**	−**0.31, −0.05**	−**0.178**	−**2.706**	**0.007**	**23 %**
**DACOBS**	−**0.660**	−**1.09, −0.23**	−**0.213**	−**3.020**	**0.003**	**10 %**
MADRS	−0.120	−0.27, 0.03	−0.104	−1.557	0.121	21 %
GFS	0.009	−0.01, 0.03	0.060	0.974	0.331	27 %
GFR	0.021	−0.002, 0.04	0.113	1.825	0.070	27 %
SANS	−0.155	−0.32, 0.01	−0.122	−1.818	0.071	18 %
**SOFAS**	**0.175**	**0.001, 0.35**	**0.121**	**1.988**	**0.048**	**29 %**
**AQoL**	−**0.246**	−**0.49, −0.01**	−**0.147**	−**2.034**	**0.043**	**12 %**
Association between WAI-C rated at 6-month and 12-month into treatment changes						
CAARMS	−0.138	−0.55, 0.27	−0.056	−0.669	0.505	27 %
**BPRS**	−**0.294**	−**0.52, −0.07**	−**0.234**	−**2.645**	**0.009**	**25 %**
DACOBS	−0.661	−1.38, 0.05	−0.176	−1.837	0.07	15 %
**MADRS**	−**0.415**	−**0.64, −0.19**	−**0.290**	−**3.587**	**<0.001**	**36 %**
GFS	0.014	−0.01, 0.04	0.078	1.000	0.320	38 %
GFR	0.015	−0.02, 0.05	0.064	0.835	0.406	39 %
**SANS**	−**0.299**	−**0.53, −0.07**	−**0.212**	−**2.552**	**0.012**	**31 %**
SOFAS	0.151	−0.11, 0.41	0.103	1.160	0.249	21 %
**AQoL**	−**0.611**	−**1.0, −0.22**	−**0.276**	−**3.081**	**0.003**	**31 %**

*Note:* WAI-C - Working Alliance Inventory rated by participants; CAARMS - Comprehensive Assessment of At-Risk Mental States; BPRS - Brief Psychiatric Rating Scale; DACOBS - Davos Assessment of Cognitive Biases Scale; MADRS - Montgomery-Åsberg Depression Rating Scale; GFS - Global Functioning: Social scale; GFR - Global Functioning: Role scale; SANS - Scale for the Assessment of Negative Symptoms; SOFAS - Social and Occupational Functioning Assessment Scale; AQoL - Assessment of Quality of Life. Values in bold are statistically significant.

**Table 4:** Regression analysis summary. Association between **WAI-T total** [predictor] and outcome variables, controlled for gender, age, treatment type (Step 2 or Step 3) and baseline.

Outcome	*B*	95 % CI	*β*	*t*	*p*	adj *R*^*2*^

Association between WAI-T rated at 6-month and 6-month into treatment changes						
CAARMS	−0.457	−0.97, 0.05	−0.128	−1.766	0.079	24 %
BPRS	0.153	−0.07, 0.38	0.101	1.343	0.181	23 %
DACOBS	−0.302	−1.13, 0.53	−0.06	−0.721	0.472	7 %
MADRS	−0.02	−0.28, 0.24	−0.012	−0.154	0.878	19 %
GFS	−0.02	−0.05, 0.02	−0.08	−1.045	0.298	26 %
GFR	−0.003	−0.04, 0.04	−0.01	−0.149	0.882	33 %
SANS	0.084	−0.2, 0.37	0.04	0.589	0.557	23 %
SOFAS	0.114	−0.21, 0.44	0.05	0.70	0.485	33 %
**AQoL**	**0.496**	**0.04, 0.96**	**0.177**	**2.137**	**0.034**	**9 %**
Association between WAI-T rated at 6-month and 12-month into treatment changes						
CAARMS	0.019	−0.78, 0.81	0.01	0.048	0.962	20 %
BPRS	−0.037	−0.48, 0.4	−0.02	−0.169	0.866	13 %
DACOBS	−0.522	−1.91, 0.86	−0.08	−0.752	0.455	7 %
MADRS	−0.254	−0.72, 0.21	−0.10	−1.082	0.282	28 %
GFS	−0.02	−0.07, 0.04	−0.06	−0.644	0.522	39 %
GFR	0.004	−0.07, 0.08	0.01	0.119	0.905	40 %
SANS	0.049	−0.42, 0.52	0.02	0.208	0.835	27 %
SOFAS	−0.04	−0.54, 0.47	−0.02	−0.148	0.882	21 %
AQoL	0.049	−0.75, 0.84	0.01	0.122	0.904	19 %

*Note:* WAI-T - Working Alliance Inventory rated by therapists; CAARMS - Comprehensive Assessment of At-Risk Mental States; BPRS - Brief Psychiatric Rating Scale; DACOBS - Davos Assessment of Cognitive Biases Scale; MADRS - Montgomery-Åsberg Depression Rating Scale; GFS - Global Functioning: Social scale; GFR - Global Functioning: Role scale; SANS - Scale for the Assessment of Negative Symptoms; SOFAS - Social and Occupational Functioning Assessment Scale; AQoL - Assessment of Quality of Life. Values in bold are statistically significant.

## Appendix A. Supplementary data

Supplementary data to this article can be found online at https://doi.org/10.1016/j.schres.2025.07.031.
