# Supplementary material for: Therapy process factors in early psychosis: The effect of working alliance on clinical symptoms and cognitive biases in Ultra High Risk young people
Source: Schizophr Res. Author manuscript; Available in PMC 2026 Aug 11. (PMC13456984; doi:10.1016/j.schres.2025.07.031)
Supplement: Supplementary file 1 [file NIHMS2180510-supplement-1.pdf]

Table 1. Operationalized UHR intake criteria.

**1. Group 1: Trait Vulnerability Group**
Family history of psychosis in first degree relative OR Schizotypal Personality Disorder (as defined by DSM IV) in identified patient
AND
**Drop in Functioning:**
Recency: Change in functioning occurred within last year
Impact: SOFAS score at least 30% below previous level of functioning and sustained for at least one month.
OR
**Sustained low functioning:**
Recency: For the past 12 months or longer
Impact: SOFAS score of 50 or less.
**2. Group 2: Attenuated Psychotic Symptoms Group**
**2a) Subthreshold intensity:**
Intensity: Global Rating Scale Score of 3-5 on Unusual Thought Content subscale, 3-5 on Non-Bizarre Ideas subscale, 3-4 on Perceptual Abnormalities subscale and/or 4-5 on Disorganised Speech subscales of the CAARMS
Frequency: Frequency Scale Score of 3-6 on Unusual Thought Content, Non-Bizarre Ideas, Perceptual Abnormalities and/or Disorganised Speech subscales of the CAARMS
Duration: symptoms present for at least one week
Recency: symptoms present in past year
**2b) Subthreshold frequency:**
Intensity: Global Rating Scale Score of 6 on Unusual Thought Content subscale, 6 on Non-Bizarre Ideas subscale, 5-6 on Perceptual Abnormalities subscale and/or 6 on Disorganised Speech subscales of the CAARMS
Frequency: Frequency Scale Score of 3 on Unusual Thought Content, Non-Bizarre Ideas, Perceptual Abnormalities and/or Disorganised Speech subscales of the CAARMS
Recency: symptoms present in past year
**3. Group 3: BLIPS Group**
Intensity: Global Rating Scale Score of 6 on Unusual Thought Content subscale, 6 on Non-Bizarre Ideas subscale, 5 or 6 on Perceptual Abnormalities subscale and/or 6 on Disorganised Speech subscales of the CAARMS
Frequency: Frequency Scale Score of 4-6 on Unusual Thought Content, Non-Bizarre Ideas, Perceptual Abnormalities and/or Disorganised Speech subscales
Duration: Symptoms present for less than one week and spontaneously remit on every occasion.
Recency: symptoms present in past year

Reference: Nelson, B., Amminger, G. P., Yuen, H. P., Wallis, N., J Kerr, M., Dixon, L., Carter, C., Loewy, R., Niendam, T. A., Shumway, M., Morris, S., Blasioli, J., & McGorry, P. D. (2018). Staged Treatment in Early Psychosis: A sequential multiple assignment randomised trial of interventions for ultra high risk of psychosis patients. Early intervention in psychiatry, 12(3), 292–306. https://doi.org/10.1111/eip.12459

[SUPPLEMENTARY] Table 2. Correlations between <u>6-month changes</u> and working alliance assessed at 6- and 12-month follow-up.

	1	2	3	4	5	6	7	8	9	10	11	12
1. WAIC-6	.	.	.	.	.	.	.	.	.	.	.	.
2. WAIT-6	**0.272*** **	.	.	.	.	.	.	.	.	.	.	.
3. WAIC-12	**0.635*** **	0.202	.	.	.	.	.	.	.	.	.	.
4. WAIT-12	**0.337* **	**0.711*** **	**0.421** **	.	.	.	.	.	.	.	.	.
5. CAARMS	**-0.149* **	-0.079	-0.070	-0.175	.	.	.	.	.	.	.	.
6. BPRS	-0.097	0.155	0.067	0.203	**0.462*** **	.	.	.	.	.	.	.
7. DACOBS	**-0.209** **	-0.017	-0.192	0.025	**0.332*** **	**0.366*** **	.	.	.	.	.	.
8. MADRS	-0.030	-0.016	-0.082	-0.070	**0.293*** **	**0.622*** **	**0.312*** **	.	.	.	.	.
9. GFS	0.038	-0.136	-0.065	-0.019	**-0.155* **	**-0.312*** **	-0.103	**-0.256*** **	.	.	.	.
10. GFR	0.056	-0.053	-0.033	-0.219	**-0.155* **	**-0.259*** **	-0.087	**-0.180* **	**0.286*** **	.	.	.
11. SANS	-0.044	0.104	-0.041	0.117	**0.206** **	**0.561*** **	**0.186* **	**0.524*** **	**-0.434*** **	**-0.443*** **	.	.
12. SOFAS	0.107	0.015	0.099	-0.088	**-0.162* **	**-0.316*** **	**-0.170* **	**-0.301*** **	**0.425*** **	**0.622*** **	**-0.479*** **	.
13. AQoL	-0.058	**0.192* **	-0.185	-0.002	**0.227** **	**0.448*** **	**0.424*** **	**0.635*** **	**-0.234** **	**-0.201** **	**0.357*** **	**-0.297*** **

[SUPPLEMENTARY] Table 3. Correlations between <u>12-month changes</u> and working alliance assessed at 6- and 12-month follow-up.

	1	2	3	4	5	6	7	8	9	10	11	12
1. WAIC-6	.	.	.	.	.	.	.	.	.	.	.	.
2. WAIT-6	**0.272*** **	.	.	.	.	.	.	.	.	.	.	.
3. WAIC-12	**0.635*** **	0.202	.	.	.	.	.	.	.	.	.	.
4. WAIT-12	**0.337* **	**0.711*** **	**0.421** **	.	.	.	.	.	.	.	.	.
5. CAARMS	-0.124	-0.002	-0.052	-0.123	.	.	.	.	.	.	.	.
6. BPRS	**-0.172* **	0.118	-0.141	0.036	**0.535*** **	.	.	.	.	.	.	.
7. DACOBS	**-0.221* **	-0.071	**-0.258* **	-0.101	**0.338*** **	**0.352*** **	.	.	.	.	.	.
8. MADRS	**-0.181* **	-0.057	**-0.229* **	-0.146	**0.285*** **	**0.646*** **	**0.335*** **	.	.	.	.	.
9. GFS	0.080	-0.101	0.054	0.075	**-0.229** **	**-0.433*** **	-0.115	**-0.345*** **	.	.	.	.
10. GFR	-0.004	-0.058	-0.013	-0.103	-0.137	**-0.240** **	**-0.217* **	-0.129	**0.292*** **	.	.	.
11. SANS	-0.096	0.121	-0.126	0.045	**0.302*** **	**0.644*** **	**0.290*** **	**0.610*** **	**-0.506*** **	**-0.408*** **	.	.
12. SOFAS	0.072	0.113	0.138	0.062	-0.129	**-0.307*** **	**-0.288*** **	**-0.296*** **	**0.461*** **	**0.590*** **	**-0.500*** **	.
13. AQoL	**-0.182* **	0.068	**-0.374** **	0.015	**0.350*** **	**0.549*** **	**0.504*** **	**0.627*** **	**-0.273** **	**-0.242** **	**0.489*** **	**-0.384*** **

Note: WAI-C - Working Alliance Inventory rated by Client; CAARMS - Comprehensive Assessment of At-Risk Mental States; BPRS - Brief Psychiatric Rating Scale; DACOBS - Davos Assessment of Cognitive Biases Scale; MADRS - Montgomery-Åsberg Depression Rating Scale; GFS - Global Functioning: Social scale; GFR - Global Functioning: Role scale; SANS - Scale for the Assessment of Negative Symptoms; SOFAS - Social and Occupational Functioning Assessment Scale; AQoL - Assessment of Quality of Life; * < 0.05; ** < 0.01; *** < 0.001
